# Metabolic syndrome in relation to risk of meningioma

**DOI:** 10.18632/oncotarget.13667

**Published:** 2016-11-26

**Authors:** Corinna Seliger, Christoph R Meier, Claudia Becker, Susan S Jick, Martin Proescholdt, Ulrich Bogdahn, Peter Hau, Michael F Leitzmann

**Affiliations:** ^1^ Department of Neurology and Wilhelm Sander-NeuroOncology Unit, Regensburg University Hospital, Regensburg, Germany; ^2^ Basel Pharmacoepidemiology Unit, Division of CIinical Pharmacy and Epidemiology, Department of Pharmaceutical Sciences, University of Basel, Basel, Switzerland; ^3^ Boston Collaborative Drug Surveillance Program, Boston University School of Public Health, Boston University, MA, USA; ^4^ Hospital Pharmacy, University Hospital Basel, Basel, Switzerland; ^5^ Department of Neurosurgery, Regensburg University Hospital, Regensburg, Germany; ^6^ Department of Epidemiology and Preventive Medicine, University of Regensburg, Regensburg, Germany

**Keywords:** meningioma, epidemiology, case-control study, metabolic syndrome

## Abstract

**Background:**

Meningioma is a frequent primary intracranial tumor, the etiology of which is potentially related to adiposity. Metabolic syndrome (MetS) is an increasingly common disease characterized by having at least three of the following conditions: central adiposity, arterial hypertension, dyslipidemia, and insulin resistance. Only one prior study investigated MetS in relation to meningioma risk and found a positive association between the two.

**Results:**

Among 2,027 cases and 20,269 controls, body mass index was positively associated with meningioma (*p-value* for trend < 0.0001). Arterial hypertension was also associated with an increased risk of meningioma (OR = 1.34; 95% CI = 1.20– 1.49). By comparison, high-density lipoprotein, triglycerides, fasting serum glucose, and use of ACE-inhibitors, AT-II inhibitors, beta-blockers, diuretics, calcium antagonists, nitrates, or statins were not associated with risk of meningioma.

**Materials and Methods:**

We conducted a matched case-control analysis using data from the U.K.-based Clinical Practice Research Datalink (CPRD) to analyse medical conditions and treatments related to MetS in cases with meningioma and meningioma-free controls. We identified all cases with an incident diagnosis of meningioma between 1995 and 2015 and matched each to ten controls on age, sex, calendar time, general practice, and number of years of active history in the CPRD prior to the index date. Exposures were assessed using computerised records. We conducted conditional logistic regression analysis to determine relative risks, estimated as odds ratios (ORs) with 95% confidence intervals (CIs), adjusted for confounding factors.

**Conclusions:**

Obesity and arterial hypertension are positively associated with risk of meningioma. Further studies are needed to better understand potential underlying biologic mechanisms.

## INTRODUCTION

Meningioma is the most common primary intracranial tumor, accounting for 36.4% of all central nervous system (CNS) tumors and about 53.4% of all benign CNS tumors [1]. The incidence of meningioma increases sharply after age 65 years and it affects women more frequently than men [1]. Risk factors for meningioma are poorly defined and include a history of ionizing radiation and rare familial cancer syndromes [2]. In addition, female sex hormones [3, 4] are thought to promote meningioma development.

According to the Adult Treatment Panel III definition [5], metabolic syndrome (MetS) affects between 7 to 57% of individuals worldwide [6] and it is characterised by having at least three of the following conditions: central adiposity (waist circumference > 102 cm (men) or > 88 cm (women)), arterial hypertension (≥ 130/85 mmHg), dyslipidemia (HDL cholesterol: < 40 mg/ dl (men) or < 50 mg/dl (women) or triglycerides (TAG) (≥ 150 mg/ dl), and impaired glucose tolerance (fasting glucose ≥ 110 mg/dl) [5–7]. MetS frequently culminates in cardiovascular diseases, which are major causes of morbidity and mortality [7]. Only one previous cohort study systematically quantified the relation between MetS and the risk of brain tumors and reported a statistically significantly increased risk of meningioma in patients with MetS (HR = 1.31; 95% CI = 1.11–1.54) [8]. Numerous studies investigated individual components of MetS, such as arterial hypertension [9], impaired glucose tolerance/diabetes [9–12], or dyslipidaemia [13, 14] and showed positive [9, 12–14] or null associations with meningioma risk [10, 11]. A number of cohort [15–19] and case-control [20, 21] studies examined the association between adiposity and meningioma, the results of which were summarized in a recent meta-analysis that reported a positive relation between the two [22].

Possible etiologic pathways linking MetS to increased risk of meningioma include increased insulin signalling [23, 24] and chronic low-grade inflammation [25, 26] present in adiposity. Despite plausible underlying biological mechanisms, comprehensive data evaluating all MetS components in relation to meningioma risk are sparse, prompting us to perform the current study.

## RESULTS

We identified 2,027 meningioma cases and 20,269 controls in the CPRD. The mean age ± standard deviation (SD) at the index date was 61.6 ± 15.2 years. A total of 75.7% of cases were women. The mean number of years of active history in the database prior to the index date was 11.2 ± 5.0 years for cases and controls.

Characteristics of meningioma cases and controls are shown in Table 1. Current smoking was associated with a slightly reduced risk of meningioma, whereas past smoking showed a significantly inverse relation to meningioma. Similarly, a history of myocardial infarction (MI) was associated with reduced risk of meningioma (OR = 0.67; 95% CI = 0.49–0.91), whereas use of estrogens was associated with a significant but weakly increased risk of meningioma, which was also not dependent on duration of drug use (OR for 1–8 prescriptions = 1.31; 95% CI = 1.09– 1.57; OR for ≥ 9 prescriptions = 1.12; 95% CI = 0.94–1.34). By comparison, no associations with meningioma were found for history of stroke, congestive heart failure (CHF), or diabetes.

**Table 1:** Characteristics of meningioma cases and controls

Variable	Number of cases(%)(*n*=2,027)	Number of controls(%)(*n*=20,269)	OR (95% CI)*	*p*-value
Age (years)*				
0–9	1 (0.1)	10 (0.1)	-	-
10–19	17 (0.8)	173 (0.9)	-	-
20–29	24 (1.2)	261 (1.3)	-	-
30–39	117 (5.8)	1,161 (5.7)	-	-
40–49	301 (14.9)	3,018 (14.9)	-	-
50–59	396 (19.5)	3,963 (19.6)	-	-
60–69	455 (22.5)	4,526 (22.3)	-	-
70–79	461 (22.7)	4,638 (22.9)	-	-
80–90	255 (12.6)	2,519 (12.4)		
Sex*				
Women	1,534 (75.7)	15,340 (75.7)	-	-
Men	493 (24.3)	4,929 (24.3)	-	-
**Smoking status**				
Never smoker	1,103 (54.4)	10,247 (50.6)	1.00 (referent)	-
Current smoker	308 (15.2)	3,249 (16.0)	0.87 (0.76–1.00)	0.050
Past smoker	450 (22.2)	4,782 (23.6)	0.87 (0.77–0.98)	***0.024***
Unknown	166 (8.2)	1,991 (9.8)	0.74 (0.61–0.89)	*0.002*
**Comorbidities**				
Stroke/TIA	89 (4.4)	958 (4.7)	0.92 (0.73–1.16)	0.481
MI	45 (2.2)	656 (3.2)	0.67 (0.49–0.91)	***0.011***
CHF	43 (2.12)	449 (2.5)	0.85 (0.61–1.18)	0.326
Diabetes	145 (7.2)	1,465 (7.2)	0.99 (0.82–1.19)	0.899
**Comedications**				
Estrogens^+^				
No prior use	1,182 (77.1)	12,196 (79.5)	1.00 (reference)	-
1–8 Rx	162 (10.6)	1,323 (8.6)	1.31 (1.09–1.57)	***0.004***
≥ 9 Rx	190 (12.4)	1,821 (11.9)	1.12 (0.94–1.34)	0.206

* Matching variables: age, sex, general practice, and number of years of active history in the database.

+ Women only.

TIA: transient ischemic attack, MI: myocardial infarction; CHF: congestive heart failure; OR: odds ratio CI: confidence intervals.

We investigated MetS-associated conditions in relation to risk of meningioma (Table 2). As compared with normal weight, obesity (OR = 1.33; 95% CI = 1.17–1.52) was associated with an increased risk of meningioma, whereas underweight was associated with significantly reduced risk of meningioma (OR = 0.63; 95% CI = 0.40–0.99). The trend for increasing body mass index (BMI) in relation to meningioma risk was highly statisticallysignificant (*p* < 0.0001). Arterial hypertension was also related to increased risk of meningioma (OR = 1.34; 95% CI = 1.20– 1.49), which was supported by statistically significant tests for trend for increasing values of systolic and diastolic blood pressure (*p-value* for trend for SBP = 0.040; *p-value* for trend for DBP = 0.013). Longer duration of arterial hypertension was also associated with significantly increased risk of meningioma (OR for 3–5.9 years versus < 3 years = 1.22; 95% CI = 1.01– 1.48, OR for ≥ 6 years versus < 3 years = 1.34; 95% CI = 1.18–1.52, *p-value* for trend = < 0.0001). There were no significant relations between coding for dyslipidemia, duration of dyslipidemia (*p-value* for trend = 0.236), HDL, TAG, or FSG and risk of meningioma. After adjusting all components of MetS for each other, the relations of obesity and arterial hypertension to meningioma risk remained minimal but statistically significant (OR for obesity = 1.28; 95% CI = 1.11–1.46; OR for arterial hypertension = 1.26; 95% CI = 1.13–1.42). When we calculated a combined metabolic syndrome variable, only the combination of hypertension, obesity, and increased TAG in women was statistically significant (OR for combined metabolic syndrome variable for women = 1.44; 95% CI = 1.17–1.78; OR for combined metabolic syndrome variable for men = 1.11; 95% CI = 0.68–1.84).

**Table 2:** Risk of meningioma in relation to conditions of metabolic syndrome

Variable	Number of cases(%)(*n*=2,027)	Number of controls(%)(*n*=20,269)	Adjusted OR (95% CI)*
**BMI (kg/m^2^)**			
< 18.5	21 (1.04)	358 (1.8)	***0.63 (0.40–0.99)***
18.5–24.9	630 (31.1)	6,710 (33.1)	1.00 (reference)
25.0–29.9	611 (30.1)	5,825 (28.7)	1.13 (1.00–1.27)
≥ 30.0	439 (21.7)	3,624 (17.9)	***1.33 (1.17–1.52)***
Unknown	326 (16.1)	3,752 (18.5)	0.94 (0.79–1.11)
p-value for trend			***< 0.0001***
**Arterial hypertension**	699 (34.5)	5,960 (29.4)	***1.34 (1.20–1.49)***
**Systolic blood pressure**			
< 120 mmHg	294 (14.5)	3,142 (15.5)	1.00 (reference)
120–139 mmHg	769 (37.9)	7,571 (37.4)	1.11 (0.96–1.29)
140–159 mmHg	637 (31.4)	5,979 (29.5)	1.18 (1.00–1.38)
160–179 mmHg	167 (8.2)	1,638 (8.1)	1.13 (0.91–1.40)
≥ 180 mmHg	48 (2.4)	448 (2.2)	1.19 (0.85–1.66)
Unknown	112 (5.5)	1,491 (7.4)	0.79 (0.59–1.06)
*p*-value for trend			***0.040***
**Diastolic blood pressure**			
< 80 mmHg	755 (37.3)	8,108 (40.0)	1.00 (reference)
80–89 mmHg	852 (42.0)	7,930 (39.1)	1.16 (1.05–1.29)
90–99 mmHg	251 (12.4)	2,236 (11.0)	***1.23 (1.05–1.43)***
≥ 100 mHg	57 (2.8)	504 (2.5)	1.25 (0.94–1.66)
Unknown	112 (5.5)	1,491 (7.4)	0.80 (0.61–1.05)
*p*-value for trend			***0.013***
**Dyslipidemia**	228 (11.3)	2,110 (10.4)	1.13 (0.96–1.32)
**HDL-cholesterol**			
≥ 60 mg/dl	274 (13.5)	2,653 (13.1)	1.00 (reference)
40–59 mg/dl	357 (17.6)	3,239 (16.0)	1.08 (0.91–1.28)
< 40 mg/dl	91 (4.5)	851 (4.2)	1.08 (0.83–1.40)
Unknown	1,305 (64.4)	13,526 (66.7)	0.88 (0.75–1.04)
*p*-value for trend			0.287
**Triglycerides**			
< 150 mg/dl	471 (23.2)	4,387 (21.6)	1.00 (reference)
150–199 mg/dl	136 (6.7)	1,257 (6.2)	1.01 (0.83–1.24)
≥ 200 mg/dl	137 (6.8)	1,388 (6.9)	0.93 (0.76–1.14)
Unknown	1,283 (63.3)	13,237 (65.3)	0.86 (0.75–0.98)
*p*-value for trend			0.535
**Serum glucose+**			
< 100 mg/dl	219 (10.8)	2,019 (10.0)	1.00 (reference)
100–126 mg/dl	68 (3.4)	627 (3.1)	1.01 (0.76–1.35)
≥ 126 mg/dl	45 (2.2)	460 (2.3)	0.91 (0.65–1.28)
Unknown	1,695 (83.6)	17,163 (84.7)	0.88 (0.73–1.05)
*p*-value for trend			0.547

* Matching variables: age, sex, general practice, and number of years of active history in the database. All analyses were adjusted for smoking, history of myocardial infarction, and estrogen use (among women).

+ Serum glucose was fasting.

We next examined use of medications prescribed for components of MetS, including antihypertensive drugs and statins (Table 3). Use of angiotensin converting enzyme inhibitors (ACE-inhibitors), angiotensin-II receptor inhibitors (AT II-inhibitors), beta-blockers, diuretics, calcium antagonists, nitrates, or statins was not associated with meningioma risk. Without adjustment for BMI, 10–29 prescriptions of diuretics and 10– 29 prescriptions of beta-blockers were positively associated with a small risk of meningioma (OR = 1.21; 95% CI = 1.01–1.46; OR = 1.28; 95% CI = 1.05–1.56, respectively).

**Table 3:** Risk of meningioma in patients using medications to treat metabolic syndrome

Variable	Number of cases (%)(*n*=2,027)	Number of controls(%) (*n* = 20,269)	Adjusted OR (95% CI)*
**ACE-inhibitors**			
No prior use	1,662 (82.0)	16,843 (83.1)	1.00 (reference)
2–9 Rx	118 (5.8)	932 (4.6)	1.10 (0.88–1.37)
10–29 Rx	103 (5.1)	964 (4.8)	0.96 (0.76–1.20)
≥ 30 Rx	144 (7.1)	1,530 (7.6)	0.89 (0.72–1.10)
*p*-value for trend			0.211
**AT II-inhibitors**			
No prior use	1,892 (93.3)	19,206 (94.8)	1.00 (reference)
2–9 Rx	32 (1.6)	258 (1.3)	1.11 (0.75–1.62)
10–29 Rx	52 (2.6)	378 (1.9)	1.22 (0.89–1.66)
≥ 30 Rx	51 (2.5)	427 (2.1)	1.10 (0.79–1.52)
*p*-value for trend			0.413
**Beta-blockers**			
No prior use	1,516 (74.8)	15,797 (77.9)	1.00 (reference)
2–9 Rx	141 (7.0)	1,227 (6.1)	1.17 (0.96–1.41)
10–29 Rx	138 (6.8)	1,108 (5.5)	***1.27 (1.04–1.54)***
≥ 30 Rx	232 (11.5)	2,137 (10.5)	1.16 (0.98–1.37)
*p*-value for trend			0.105
**Diuretics**			
No prior use	1,337 (66.0)	13,949 (69.8)	1.00 (reference)
2–9 Rx	74 (3.7)	675 (3.3)	1.12 (0.92–1.35)
10–29 Rx	170 (8.4)	1,598 (7.9)	1.17 (0.97–1.41)
≥ 30 Rx	446 (22.0)	4,047 (20.0)	1.04 (0.88–1.23)
*p*-value for trend			0.946
**Calcium antagonists**			
No prior use	1,642 (81.0)	16,778 (82.8)	1.00 (reference)
2–9 Rx	112 (5.5)	932 (4.6)	1.11 (0.90–1.39)
10–29 Rx	105 (5.2)	967 (4.8)	1.02 (0.81–1.27)
≥ 30 Rx	168 (8.3)	1,592 (7.9)	1.06 (0.87–1.29)
*p*-value for trend			0.670
**Nitrates**			
No prior use	1,887 (93.1)	18,708 (92.3)	1.00 (reference)
2–9 Rx	61 (3.0)	652 (3.2)	0.89 (0.67–1.18)
10–29 Rx	31 (1.5)	345 (1.7)	0.86 (0.59–1.27)
≥ 30 Rx	48 (2.4)	564 (2.8)	0.86 (0.62–1.20)
*p*-value for trend			0.389
**Statins**			
No prior use	1,693 (83.5)	16,911 (83.4)	1.00 (reference)
2–9 Rx	75 (3.7)	684 (3.4)	1.01 (0.78–1.31)
10–29 Rx	113 (5.6)	1,072 (5.3)	0.98 (0.78–1.21)
≥ 30 Rx	146 (7.2)	1,602 (7.9)	0.88 (0.71–1.09)
*p*-value for trend			0.244

* Matching variables: age, sex, general practice, and number of years of active history in the database. All analyses were adjusted for smoking, BMI, history of MI, and estrogen use (among women).

When we stratified our analyses by sex (Supplementary Tables S1 and S2), the positive associations of obesity (OR = 1.37; 95% CI = 1.18–1.58) and diastolic blood pressure (*p-value* for trend=0.049) with risk of meningioma was limited to women. Arterial hypertension was associated with increased risk of meningioma in both women (OR = 1.35; 95% CI = 1.19–1.53) and men (OR = 1.30; 95% CI = 1.04–1.62), even after additional adjustment for BMI (OR for arterial hypertension in men = 1.28; 95% CI = 1.02–1.60; OR for arterial hypertension in women = 1.27; 95% CI = 1.12–1.45).

## DISCUSSION

Our matched case-control analysis revealed positive associations between certain components of MetS, adiposity and arterial hypertension, and risk of meningioma. By comparison, dyslipidemia and impaired glucose tolerance were not related to meningioma risk nor were use of antihypertensive drugs or statins.

A previous cohort study investigated the relation of metabolic syndrome to the risk of brain tumors and reported a minor but statistically significant increased risk of meningioma in patients suffering from MetS (hazard ratio (HR) derived from MetS z-score = 1.31, 95% CI = 1.11–1.54) [8]. In that study, both increased SBP and DBP were associated with increased meningioma risk (HR for SBP = 1.27 per unit standard deviation; 95% CI = 1.03–1.57; HR for DBP = 1.29, 95% CI = 1.04–1.58), whereas the effect estimates for BMI, TAG, HDL, and glucose levels were not related to meningioma risk. In contrast to that study, our analysis was based on a larger number of meningioma cases (2,027 versus 348 meningioma cases) and we additionally explored specific medications used to treat individual components of metabolic syndrome.

Numerous cohort [3, 16–19] and case-control [20, 21] studies explored the relation between adiposity and meningioma, and a recent meta-analysis reported an increased risk of meningioma in overweight and adipose individuals [22]. Obesity is associated with increased circulating levels of insulin [23] and insulin-like growth factor [24] as well as excess production of estrogens in adipose tissue [27, 28], both of which may promote meningioma development [29–32], especially in obese women, to whom the positive risk association was limited in our study. Chronic low-grade inflammation with impaired immune function [25, 26], increased oxidative stress, and decreased antioxidant defence mechanisms [33] associated with metabolic syndrome may also contribute to the development of meningioma.

The inverse relation of meningioma to smoking, a risk factor for coronary heart disease, may partly explain the inverse association between meningioma and myocardial infarction in our study.

A case-control study based on 306 patients with meningioma reported that arterial hypertension was positively associated with the incidence of meningioma in women in the 60–69 year age group (OR = 2.23; 95% CI = 1.03–4.84) [9]. We found an increased risk of meningioma with arterial hypertension even after controlling for BMI, suggesting that hypertension reflects metabolic pathways that exert an effect on meningioma development independently from mechanisms related to obesity. We do not think that reverse causation due to increased intracranial pressure caused by meningioma growth is responsible for the positive association observed with arterial hypertension in our study. Increased intracranial pressure may lead to increased systemic blood pressure as an adaptation mechanism to provide a stable intracranial blood flow against higher resistance, which is also known as “Cushing reflex” [34]. However, not all meningiomas cause sufficiently increased intracranial pressure to induce a cushing response, and increased intracranial pressure usually evolves slowly, arguing against reverse causation. Additionally, many other intracranial tumors are not associated with hypertension.

Although increased insulin signalling may play a role in meningioma formation, impaired glucose tolerance and diabetes were not associated with meningioma in our study. Prior investigations found both positive [9, 12], null [10, 11] and inverse [35] associations between diabetes and risk of meningioma. One previous study specifically investigated risk of meningioma in patients with impaired glucose tolerance and found no association between the two [8]. That study did also not find a statistically significant association between cholesterol, TAG and meningioma risk [8]. Another recent study found an inverse association between elevated FSG and meningioma risk among women and with increased serum cholesterol within the year before diagnosis for both men and women [35]. In our study, HDL and TAG were not related to risk of meningioma, although dyslipidemia showed a borderline positive association with risk of meningioma in women. However, our data regarding FSG, TAG and HDL contained large numbers of unknowns, which may have restricted our power to fully analyse those variables.

Certain limitations of our study need to be discussed. Our study may be influenced by detection bias, because patients with hypertension who are on many medications may be monitored more closely, which may lead to diagnosis of non-symptomatic (or less severe) meningiomas. Also, we may have underestimated the number of meningioma cases due to subclinical tumors that were not detected on imaging. Therefore our study might have been enriched for patients with meningiomas in functionally important brain areas or with larger tumor volume. We were not able to control for exposure to ionizing radiation, the only known modifiable risk factor for meningioma [36]. However, the proportion of meningioma cases due to radiation is small, and we excluded patients with cancers other than non-melanoma skin cancer. We were also not able to control for socioeconomic status and education level, but we matched our cases and controls by general practice, which at least partially controls for socioeconomic status by choosing comparable neighbourhoods. We could not take lifestyle factors such as physical activity into account in our analyses, which may influence meningioma risk [15, 22]. Unfortunately, we were not able to stratify meningiomas according to their degree of malignancy.

Our study has several important strengths. We were able to study meningioma in relation to BMI, arterial hypertension, and dyslipidemia, supported by measurements for SBP and DBP, as well as HDL, TAG, and fasting serum glucose. Additionally, we investigated medications used to treat conditions of metabolic syndrome and we stratified our main results by sex. The CPRD is a well-established, large, and validated database [37, 38] reporting on a population with free access to health services, thereby ensuring broad generalizability. Cases and controls were generated from a pre-existing database, therefore selection bias was minimized. Further, recall bias was absent because the data regarding medications and concomitant diseases were collected prospectively and routinely, free of any study hypothesis. We further shifted the index date by three years to account for various potential biases such as increased diagnostic procedures, comorbidities in patients with meningioma, or changes in adherence to medication use due to cognitive impairment of thus far undiagnosed meningioma. Finally, we restricted our study to patients with an active history of at least three years in the CPRD database prior to the index date in order to increase the probability of capturing only incident cases, as well as to make sure that study participants had sufficiently long histories of exposure to conditions of metabolic syndrome.

In summary, obesity and arterial hypertension were associated with a marginally increased risk of meningioma in this large population-based analysis using data from a well-validated primary care database. In consideration of the limited number of modifiable risk factors for meningioma, preventive strategies should consider including weight control and blood pressure normalization, which may influence disease development. In addition, the threshold for obtaining brain imaging studies for obese women and men with hypertension who present with headache or other signs of increased intracranial pressure should possibly be lowered.

## MATERIALS AND METHODS

### Data source

The Clinical Practice Research Datalink (CPRD) is a primary care database which encompasses patient information from around 8.5% of the population of the United Kingdom (U.K.). Participating general practices are representative of the U.K. general population with respect to age, sex, and ethnicity. The U.K. National Health Service guarantees universal coverage, so no part of the population is excluded. General practitioners are trained to code demographic data, physical findings, symptoms, diagnoses, referrals, hospital admissions, drug prescriptions, and deaths in an anonymous format using standard coding systems [37]. The diagnostic coding in the CPRD has been extensively validated [38, 39]. The current study was approved by the Independent Scientific Advisory Committee of the CPRD (protocol-number: 16_122) and made available to the journal reviewers.

### Study population

### Case definition

We defined cases as patients in the CPRD with newly diagnosed meningioma between 1995 and 2015. Meningioma diagnoses were based on specific medical READ codes (Supplementary Table S3).

The date of the first meningioma diagnosis minus three years was referred to as the ‘index date’. We shifted the date of diagnosis back in time by three years for cases and matched controls to account for potential lag time between disease development and detection, to account for possible changes in medication use prior to the diagnosis date of meningioma, to account for potential earlier detection of pre-existing concomitant diseases in case patients caused by early symptoms of undiagnosed meningioma, and to be sure we captured exposure information prior to disease onset.

We only included patients with an active history in the database for at least three years prior to the index date. We excluded patients older than 90 years of age from the study population, those with a history of other prior or current cancers except non-melanoma skin cancer, and those with recorded alcoholism or human immunodeficiency virus infection prior to the index date.

### Control definition

From the study population we randomly matched up to 10 control patients without a history of meningioma to each case patient on calendar time (same index date), age (same year of birth), sex, general practice, and number of years of active history in the database prior to meningioma diagnosis. Five runs were performed to identify matching controls with increasing tolerance of ±2 years for year of birth and number of years of active history in the database to obtain the predefined number of controls. The same exclusion criteria were applied to controls as to cases.

### Exposures

We assessed presence versus absence of components of MetS (adiposity, arterial hypertension, dyslipidemia, impaired glucose tolerance) using the specific Read Codes documented in the computerized records. Patients with treatment for the specific components of MetS were not excluded. For adiposity, we evaluated body mass index (BMI; < 18.5 kg/m^2^, 18.5–24.9 kg/m^2^, 25.0–29.9 kg/m^2^, ≥ 30.0 kg/m^2^). Coding for arterial hypertension (yes/no) was supported by duration of coding for arterial hypertension (< 3 years, 3–5.9 years, ≥ 6 years), systolic blood pressure (SBP) (< 120 mmHg, 120–139 mmHg, 140–159 mmHg, 160–179 mmHg, ≥ 180 mmHg) and diastolic blood pressure (DBP) (< 80 mmHg, 80–89 mmHg, 90–99 mmHg, ≥ 100 mmHg) values. Coding for dyslipidemia (yes/no) was supported by duration of coding for dyslipidemia (< 3 years, 3–5.9 years, ≥ 6 years), values for high-density lipoprotein (HDL) (< 40 mg/ dl, 40–59 mg/dl, ≥ 60 mg/dl) and triglycerides (TAG) (< 150 mg/ dl, 150–199 mg/dl, ≥ 200 mg/dl). Insulin resistance and impaired glucose tolerance was defined using fasting serum glucose levels (FSG) (< 100 mg/dl, 100–126 mg/dl, ≥ 126 mg/dl) and coding for diabetes. We always used the last documented value prior to/excluding the index date. We also calculated a combined metabolic syndrome variable, combining coding for at least three of the following variables: arterial hypertension, BMI ≥ 30 kg/ m^2^, TAG ≥ 150 mg/dl, HDL < 40 mg/dl (men) or < 50 mg/dl (women), FSG ≥ 110 mg/dl.

In addition, we explored medical treatments for components of MetS. Specifically, we considered exposure to antihypertensive drugs (angiotensin converting enzyme inhibitors, angiotensin-II receptor blockers, beta-blockers, diuretics, calcium antagonists, nitrates) and statins, which were categorized based on the number of prescriptions prior to the index date. Subjects who received < 2 prescriptions for a study medication prior to the index date were categorized as ‘no prior use’ (reference). Users were classified into categories of 2–9, 10–29 or ≥ 30 prescriptions prior to the index date. The number of prescriptions serves as an approximation of exposure duration, since an average prescription covers 45 to 90 days of treatment, depending on the number of tablets (1 or 2) taken per day.

### Statistical analysis

We conducted conditional logistic regression analysis using SAS statistical software version 9.4 (SAS Institute Inc, Cary, NC) to determine relative risks, estimated as odds ratios (ORs) with 95% confidence intervals (CIs) for various medical conditions or treatments for MetS in relation to meningioma. In univariate analyses we investigated the relations of various potential confounders with risk of meningioma including smoking status (never, current, past, unknown), use of estrogens for hormone replacement therapy in women and presence versus absence (reference) of specific medical conditions or diseases, such as stroke, transient ischemic attack (TIA), ischemic heart disease (IHD), myocardial infarction (MI), congestive heart failure (CHF). We only included variables that altered the risk of meningioma by > 10% in the final multivariable analysis [40]. We performed one multivariable model adjusted for BMI and one model without adjustment for BMI to prevent statistical over control. Analyses were also stratified by sex and age. We conducted tests of linear trend by modeling the median value of each category of drug prescriptions, laboratory or blood pressure values as a continuous variable in the multivariable model, the coefficient for which was evaluated using a Wald test. We considered a two-sided *p-value* of < 0.05 statistically significant.

## SUPPLEMENTARY MATERIALS TABLES


