# Supplementary material for: Metabolic syndrome in relation to risk of meningioma
Source: Oncotarget. 2016 Nov 26;8(2):2284–92. doi: 10.18632/oncotarget.13667 (PMC5356799; doi:10.18632/oncotarget.13667)
Supplement: Supplementary file 1 [file oncotarget-08-2284-s001.pdf]

Metabolic syndrome in relation to risk of meningioma

Supplementary Materials

Supplementary Table S1: Risk of meningioma in relation to components of metabolic syndrome in men

Variable	Number of cases (%) (*n* = 493)	Number of controls (%) (*n* = 4,929)	Adjusted OR (95% CI)*
**BMI (kg/m²)**			
< 18.5	3 (0.6)	41 (0.8)	0.81 (0.25–2.65)
18.5–24.9	120 (24.3)	1,288 (26.1)	1.00 (reference)
25–29.9	175 (35.5)	1,653 (33.5)	1.16 (0.91–1.48)
≥ 30.0	80 (16.2)	749 (15.2)	1.19 (0.88–1.62)
Unknown	115 (23.3)	1,198 (24.3)	1.04 (0.75–1.45)
p-value for trend			0.251
**Arterial hypertension**	161 (32.7)	1,390 (28.2)	***1.30 (1.04–1.62)***
**Systolic blood pressure**			
< 120 mmHg	45 (9.1)	517 (10.5)	1.00 (reference)
120–139 mmHg	196 (39.8)	1,846 (37.5)	1.22 (0.87–1.73)
140–159 mmHg	150 (30.4)	1,513 (30.7)	1.14 (0.80–1.63)
160–179 mmHg	42 (8.5)	390 (7.9)	1.23 (0.78–1.95)
≥ 180 mmHg	9 (1.8)	84 (1.7)	1.22 (0.57–2.63)
Unknown	51 (10.3)	579 (11.8)	0.95 (0.57–1.58)
p-value for trend			0.338
**Diastolic blood pressure**			
< 80 mmHg	161 (32.7)	1,735 (35.2)	1.00 (reference)
80–89 mmHg	193 (39.2)	1,892 (38.4)	1.10 (0.88–1.38)
90–99 mmHg	70 (14.2)	582 (11.8)	1.30 (0.96–1.78)
≥ 100 mmHg	18 (3.7)	141 (2.9)	1.41 (0.83–2.40)
Unknown	51 (10.3)	579 (11.8)	0.90 (0.57–1.43)
p-value for trend			0.116
**Dyslipidemia**	54 (11.0)	565 (11.5)	0.99 (0.72–1.36)
**HDL-cholesterol**			
≥ 60 mg/dl	36 (7.3)	345 (7.0)	1.00 (reference)
40–59 mg/dl	104 (21.1)	1,000 (20.3)	0.99 (0.67–1.48)
< 40 mg/dl	49 (9.9)	438 (8.9)	1.12 (0.71–1.79)
Unknown	5 (0.2)	46 (0.2)	0.84 (0.56–1.26)
p-value for trend			0.520
**Triglycerides**			
< 150 mg/dl	106 (21.5)	1,132 (23.0)	1.00 (reference)
150–199 mg/dl	41 (8.3)	350 (7.1)	1.27 (0.87–1.87)
≥ 200 mg/dl	47 (9.5)	459 (9.3)	1.13 (0.78–1.63)
Unknown	299 (60.7)	2,988 (60.6)	1.05 (0.79–1.39)
p-value for trend			0.498
**Serum glucose⁺**			
< 100 mg/dl	50 (10.1)	416 (8.4)	1.00 (reference)
100–126 mg/dl	17 (3.5)	188 (3.8)	0.77 (0.43–1.38)
≥ 126 mg/dl	13 (2.6)	148 (3.0)	0.73 (0.38–1.40)
Unknown	413 (83.8)	4,177 (84.7)	0.77 (0.53–1.11)
p-value for trend			0.765

*Matching variables: age, sex, general practice, and number of years of active history in the database. All analyses were adjusted for smoking, BMI, history of MI, and estrogen use (among women).

⁺Serum glucose was fasting.

Supplementary Table S2: Risk of meningioma in relation to conditions of metabolic syndrome in women

Variable	Number of cases (%) (*n* = 1,535)	Number of controls (%) (*n* = 15,340)	Adjusted OR (95% CI)*
**BMI (kg/m²)**			
< 18.5	18 (1.2)	317 (2.1)	***0.61 (0.37–0.98)***
18.5–24.9	510 (33.3)	5,422 (35.4)	1.00 (reference)
25–29.9	436 (28.4)	4,172 (27.2)	1.12 (0.98–1.28)
≥ 30.0	359 (23.4)	2,875 (18.7)	***1.37 (1.18–1.58)***
Unknown	211 (13.8)	2,554 (16.7)	*0.90 (0.73–1.10)*
p-value for trend			***< 0.0001***
**Arterial hypertension**	538 (35.1)	4,570 (29.8)	***1.35 (1.19–1.53)***
**Systolic blood pressure**			
< 120 mmHg	249 (16.2)	2,625 (17.1)	1.00 (reference)
120–139 mmHg	573 (37.4)	5,725 (37.3)	1.08 (0.92–1.28)
140–159 mmHg	487 (31.8)	4,466 (29.1)	1.20 (1.00–1.43)
160–179 mmHg	125 (8.2)	1,248 (8.1)	1.10 (0.86–1.41)
≥ 180 mmHg	39 (2.5)	364 (2.4)	1.18 (0.81–1.72)
Unknown	61 (4.0)	912 (6.0)	0.72 (0.50–1.03)
p-value for trend			0.057
**Diastolic blood pressure**			
< 80 mmHg	594 (38.7)	6,373 (41.5)	1.00 (reference)
80–89 mmHg	659 (43.0)	6,038 (39.4)	***1.18 (1.05–1.33)***
90–99 mmHg	181 (11.8)	1,654 (10.8)	1.20 (1.00–1.44)
≥ 100 mmHg	39 (2.5)	363 (2.4)	1.18 (0.83–1.67)
Unknown	61 (4.0)	912 (6.0)	0.73 (0.51–1.03)
p-value for trend			***0.049***
**Dyslipidemia**	174 (11.3)	1,545 (10.1)	1.18 (0.98–1.41)
**HDL-cholesterol**			
≥ 60 mg/dl	238 (15.5)	2,308 (15.1)	1.00 (reference)
40–59 mg/dl	253 (16.5)	2,239 (14.6)	1.11 (0.92–1.34)
< 40 mg/dl	42 (2.7)	413 (2.7)	1.00 (0.71–1.42)
Unknown	1,001 (65.3)	10,380 (67.7)	0.89 (0.74–1.06)
p-value for trend			0.381
**Triglycerides**			
< 150 mg/dl	365 (23.8)	3,255 (21.2)	1.00 (reference)
150–199 mg/dl	95 (6.2)	907 (5.9)	0.94 (0.74–1.19)
≥ 200 mg/dl	90 (5.9)	929 (6.1)	0.87 (0.68–1.11)
Unknown	984 (64.2)	10,249 (66.8)	*0.81 (0.69–0.94)*
p-value for trend			0.226
**Serum glucose⁺**			
< 100 mg/dl	169 (11.0)	1,603 (10.5)	1.00 (reference)
100–126 mg/dl	51 (3.3)	439 (2.9)	1.11 (0.80–1.55)
≥ 126 mg/dl	32 (2.1)	312 (2.0)	0.99 (0.66–1.48)
Unknown	1,282 (83.6)	12,986 (84.7)	0.91 (0.74–1.11)
p-value for trend			0.552

*Matching variables: age, sex, general practice, and number of years of active history in the database. All analyses were adjusted for smoking, BMI, history of MI, and estrogen use (among women).

⁺Serum glucose was fasting.

Supplementary Table S3: READ codes for meningioma used in this study and corresponding descriptions

READ code and description	
B7F2000	Cerebral meningioma
B7F4000	Spinal meningioma
BBd..00	[M]Meningiomas
BBd0.00	[M]Meningioma NOS
BBd1.00	[M]Meningiomatosis NOS
BBd1.11	[M]Diffuse meningiomatosis
BBd1.12	[M]Multiple meningiomatosis
BBd2.00	[M]Meningioma; malignant
BBd3.00	[M]Meningotheliomatous meningioma
BBd3.11	[M]Endotheliomatous meningioma
BBd3.12	[M]Syncytial meningioma
BBd4.00	[M]Fibrous meningioma
BBd5.00	[M]Psammomatous meningioma
BBd6.00	[M]Angiomatous meningioma
BBd7.00	[M]Haemangioblastic meningioma
BBd7.11	[M]Angioblastic meningioma
BBd8.00	[M]Haemangiopericytic meningioma
BBd9.00	[M]Transitional meningioma
BBd9.11	[M]Mixed meningioma
BBdA.00	[M]Papillary meningioma
BBdz.00	[M]Meningioma NOS
